# Methyl 1-benzyl-1*H*-1,2,3-triazole-4-carboxyl­ate

**DOI:** 10.1107/S1600536810022531

**Published:** 2010-06-18

**Authors:** Chiung-Cheng Huang, Feng-Ling Wu, Yih Hsing Lo, Wen-Rong Lai, Chia-Her Lin

**Affiliations:** a Department of Chemical Engineering, Tatung University, Taipei 104, Taiwan; b Department of Natural Science, Taipei Municipal University of Education, Taipei 10048, Taiwan; c Department of Chemistry, Chung-Yuan Christian University, Chung-Li 320, Taiwan

## Abstract

In the title compound, C_11_H_11_N_3_O_2_, prepared by the [3+2] cycloaddition reaction of benzyl azide with methyl propiolate, the dihedral angle between the ring planes is 67.87 (11)°.

## Related literature

For catalytic transformations of organic alkynes mediated by ruthenium complexes, see: Naota *et al.* (1998 ▶); Bruneau & Dixneuf (1999 ▶); Trost *et al.* (2001 ▶); Chen *et al.* (2009 ▶); Cheng *et al.* (2009 ▶). For the synthesis of triazoles, see: Padwa (1976 ▶). For applications of triazoles, see: Krivopalov & Shkurko (2005 ▶).
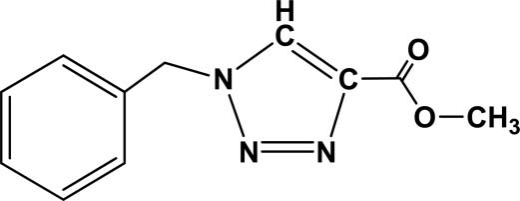


## Experimental

### 

#### Crystal data

C_11_H_11_N_3_O_2_
*M* *_r_* = 217.23
Monoclinic, 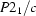

*a* = 12.0551 (6) Å
*b* = 5.6285 (3) Å
*c* = 16.7578 (10) Å
β = 110.664 (3)°
*V* = 1063.90 (10) Å^3^
*Z* = 4
Mo *K*α radiation
μ = 0.10 mm^−1^
*T* = 200 K
0.55 × 0.40 × 0.35 mm

#### Data collection

Nonuis KappaCCD diffractometer
Absorption correction: multi-scan (*SORTAV*; Blessing, 1995 ▶) *T*_min_ = 0.949, *T*_max_ = 0.967
7021 measured reflections
1845 independent reflections
1615 reflections with *I* > 2σ(*I*)
*R*_int_ = 0.023

#### Refinement

*R*[*F* ^2^ > 2σ(*F* ^2^)] = 0.034
*wR*(*F* ^2^) = 0.091
*S* = 1.01
1845 reflections
145 parameters
H-atom parameters constrained
Δρ_max_ = 0.15 e Å^−3^
Δρ_min_ = −0.16 e Å^−3^

### 

Data collection: *COLLECT* (Nonius, 1999 ▶); cell refinement: *DENZO* and *SCALEPACK* (Otwinowski & Minor, 1997 ▶); data reduction: *DENZO* and *SCALEPACK*; program(s) used to solve structure: *SHELXS97* (Sheldrick, 2008 ▶); program(s) used to refine structure: *SHELXL97* (Sheldrick, 2008 ▶); molecular graphics: *ORTEP-3* (Farrugia, 1997 ▶); software used to prepare material for publication: *WinGX* (Farrugia, 1999 ▶).

## Supplementary Material

Crystal structure: contains datablocks I, global. DOI: 10.1107/S1600536810022531/bh2294sup1.cif

Structure factors: contains datablocks I. DOI: 10.1107/S1600536810022531/bh2294Isup2.hkl

Additional supplementary materials:  crystallographic information; 3D view; checkCIF report

## Figures and Tables

**Fig. 1.**
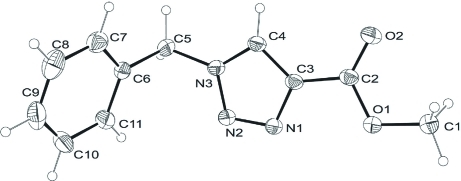
Molecular structure of the title compound with labelling and displacement ellipsoids drawn at the 30% probability level (H atoms are shown as spheres of arbitrary radius).

## supplementary crystallographic information

### Comment

Catalytic transformations of organic alkynes mediated by ruthenium complexes are well known, and confirmation for the intermediacy of ruthenium(II) acetylide and vinylidene complexes has been provided (Bruneau & Dixneuf, 1999; Cheng *et al.*, 2009; Naota *et al.*, 1998; Trost *et al.*, 2001). Therefore, ruthenium was a logical choice in our search for a new catalyst of click reaction (Chen *et al.*, 2009). Organic azides are synthetically useful reagents. Amongst many reactions, perhaps the most significant are the 1,3-dipolar cycloaddition reactions with alkynes to synthesize triazoles (Padwa, 1976). Triazoles are nitrogen heteroarenes which have found a range of important applications in the pharmaceutical and agricultural industries (Krivopalov & Shkurko, 2005).

A mixture of benzyl azide and methyl propiolate (1:1.5 equiv, respectively) in toluene was refluxed for 24 h in the presence of 5% moles of {(Tp)(PPh_3_)_2_Ru(N_3_)}, leading to the title compound [Tp is hydridotris(pyrazolyl)borate]. Single crystals of the title compound suitable for X-ray structure analysis were obtained by recrystallization of the crude product from dichloromethane–ether.

In the title compound (Fig. 1), phenyl and triazole are linked together through a methylene group. Of major interest is the methylene C atom, which presents a C—CH_2_—N angle of 112.13 (11)°, larger than the ideal tetrahedral value of 109.47°. The N3—C4, C4—C3, C3—N1, N1—N2, and N2—N3 bond lengths are 1.3367 (17), 1.3722 (17), 1.3621 (16), 1.3092 (15) and 1.3560 (15) Å, respectively, which compare with those found for C═C, N═N and C—N bonds in related compounds.

### Experimental

A mixture of benzyl azide, methyl propiolate and {(Tp)(PPh_3_)_2_Ru(N_3_)} in toluene was refluxed for 24 h. The solvent was removed under vacuum and the product was purified by silica gel chromatography. The unreacted alkyne and traces of side products were first eluted out with ether. The pure 1,4-disubstituted triazole product was then obtained by elution with CH_2_Cl_2_.

### Refinement

H atoms were placed in idealized positions and constrained to ride on their parent atoms, with C—H = 0.95–0.99 Å and *U*_iso_(H) = 1.2 *U*_eq_(C) or *U*_iso_(H) = 1.5*U*_eq_(C).

### Crystal data

**Table:**

C_11_H_11_N_3_O_2_	*F*(000) = 456
*M**_r_* = 217.23	*D*_x_ = 1.356 Mg m^−^^3^
Monoclinic, *P*2_1_/*c*	Mo *K*α radiation, λ = 0.71073 Å
Hall symbol: -P 2ybc	Cell parameters from 3680 reflections
*a* = 12.0551 (6) Å	θ = 2.6–25.0°
*b* = 5.6285 (3) Å	µ = 0.10 mm^−^^1^
*c* = 16.7578 (10) Å	*T* = 200 K
β = 110.664 (3)°	Prism, colorless
*V* = 1063.90 (10) Å^3^	0.55 × 0.40 × 0.35 mm
*Z* = 4	

### Data collection

**Table:**

Nonuis KappaCCD diffractometer	1845 independent reflections
Radiation source: fine-focus sealed tube	1615 reflections with *I* > 2σ(*I*)
graphite	*R*_int_ = 0.023
Detector resolution: 9 pixels mm^-1^	θ_max_ = 25.0°, θ_min_ = 1.8°
CCD rotation images, thick slices scans	*h* = −14→14
Absorption correction: multi-scan (*SORTAV*; Blessing, 1995)	*k* = −6→6
*T*_min_ = 0.949, *T*_max_ = 0.967	*l* = −19→18
7021 measured reflections	

### Refinement

**Table:**

Refinement on *F*^2^	Primary atom site location: structure-invariant direct methods
Least-squares matrix: full	Secondary atom site location: difference Fourier map
*R*[*F*^2^ > 2σ(*F*^2^)] = 0.034	Hydrogen site location: inferred from neighbouring sites
*wR*(*F*^2^) = 0.091	H-atom parameters constrained
*S* = 1.01	*w* = 1/[σ^2^(*F*_o_^2^) + (0.0442*P*)^2^ + 0.2782*P*] where *P* = (*F*_o_^2^ + 2*F*_c_^2^)/3
1845 reflections	(Δ/σ)_max_ < 0.001
145 parameters	Δρ_max_ = 0.15 e Å^−^^3^
0 restraints	Δρ_min_ = −0.16 e Å^−^^3^
0 constraints	

### Fractional atomic coordinates and isotropic or equivalent isotropic displacement parameters (Å^2^)

**Table:**

	*x*	*y*	*z*	*U*_iso_*/*U*_eq_	
C1	−0.11251 (12)	1.1370 (3)	0.66802 (10)	0.0462 (4)	
H1A	−0.1102	1.3069	0.6814	0.069*	
H1B	−0.1054	1.0444	0.7191	0.069*	
H1C	−0.1878	1.0991	0.6226	0.069*	
C2	−0.00887 (11)	0.8505 (2)	0.61959 (8)	0.0359 (3)	
C3	0.09145 (10)	0.8061 (2)	0.59130 (8)	0.0344 (3)	
C4	0.13667 (11)	0.5915 (2)	0.57855 (8)	0.0379 (3)	
H4	0.1102	0.4373	0.5864	0.045*	
C5	0.30857 (12)	0.4920 (2)	0.53001 (10)	0.0441 (3)	
H5A	0.3067	0.5334	0.4721	0.053*	
H5B	0.2820	0.3252	0.5288	0.053*	
C6	0.43422 (11)	0.5135 (2)	0.59233 (8)	0.0359 (3)	
C7	0.48209 (14)	0.3404 (3)	0.65326 (10)	0.0508 (4)	
H7	0.4357	0.2066	0.6564	0.061*	
C8	0.59814 (16)	0.3616 (4)	0.71015 (11)	0.0662 (5)	
H8	0.6306	0.2417	0.7518	0.079*	
C9	0.66570 (14)	0.5532 (4)	0.70663 (11)	0.0647 (5)	
H9	0.7448	0.5669	0.7456	0.078*	
C10	0.61865 (14)	0.7251 (3)	0.64659 (12)	0.0630 (5)	
H10	0.6653	0.8590	0.6442	0.076*	
C11	0.50372 (12)	0.7062 (3)	0.58917 (10)	0.0488 (4)	
H11	0.4724	0.8262	0.5474	0.059*	
N1	0.15518 (9)	0.98335 (18)	0.57253 (7)	0.0379 (3)	
N2	0.23722 (9)	0.88701 (19)	0.54855 (8)	0.0404 (3)	
N3	0.22627 (9)	0.64798 (18)	0.55266 (7)	0.0380 (3)	
O1	−0.01523 (8)	1.07905 (16)	0.63991 (6)	0.0417 (3)	
O2	−0.07666 (8)	0.69845 (17)	0.62483 (7)	0.0480 (3)	

### Atomic displacement parameters (Å^2^)

**Table:**

	*U*^11^	*U*^22^	*U*^33^	*U*^12^	*U*^13^	*U*^23^
C1	0.0442 (8)	0.0438 (8)	0.0558 (9)	−0.0033 (6)	0.0239 (7)	−0.0050 (6)
C2	0.0330 (6)	0.0314 (6)	0.0372 (7)	−0.0041 (5)	0.0047 (5)	0.0008 (5)
C3	0.0298 (6)	0.0293 (6)	0.0373 (7)	−0.0038 (5)	0.0032 (5)	−0.0005 (5)
C4	0.0326 (6)	0.0298 (6)	0.0456 (8)	−0.0047 (5)	0.0067 (6)	−0.0009 (5)
C5	0.0396 (7)	0.0351 (7)	0.0554 (8)	0.0013 (6)	0.0143 (6)	−0.0090 (6)
C6	0.0361 (7)	0.0332 (7)	0.0416 (7)	0.0016 (5)	0.0177 (6)	−0.0016 (5)
C7	0.0564 (9)	0.0444 (8)	0.0557 (9)	0.0048 (7)	0.0248 (7)	0.0087 (7)
C8	0.0688 (11)	0.0781 (13)	0.0459 (9)	0.0277 (10)	0.0131 (8)	0.0094 (8)
C9	0.0395 (8)	0.0891 (14)	0.0590 (10)	0.0112 (9)	0.0091 (7)	−0.0207 (10)
C10	0.0409 (8)	0.0662 (11)	0.0854 (13)	−0.0141 (8)	0.0266 (8)	−0.0157 (9)
C11	0.0439 (8)	0.0435 (8)	0.0618 (9)	−0.0029 (6)	0.0221 (7)	0.0054 (7)
N1	0.0323 (5)	0.0309 (6)	0.0472 (7)	−0.0012 (4)	0.0098 (5)	−0.0001 (5)
N2	0.0358 (6)	0.0300 (6)	0.0533 (7)	−0.0010 (4)	0.0134 (5)	−0.0008 (5)
N3	0.0329 (5)	0.0283 (5)	0.0474 (7)	−0.0015 (4)	0.0076 (5)	−0.0037 (5)
O1	0.0387 (5)	0.0336 (5)	0.0555 (6)	−0.0053 (4)	0.0199 (4)	−0.0067 (4)
O2	0.0437 (5)	0.0365 (5)	0.0641 (7)	−0.0083 (4)	0.0196 (5)	0.0017 (4)

### Geometric parameters (Å, °)

**Table:**

C1—O1	1.4469 (16)	C5—H5B	0.9900
C1—H1A	0.9800	C6—C7	1.3817 (19)
C1—H1B	0.9800	C6—C11	1.3830 (19)
C1—H1C	0.9800	C7—C8	1.392 (2)
C2—O2	1.2076 (15)	C7—H7	0.9500
C2—O1	1.3400 (15)	C8—C9	1.365 (3)
C2—C3	1.4678 (19)	C8—H8	0.9500
C3—N1	1.3621 (16)	C9—C10	1.367 (3)
C3—C4	1.3722 (17)	C9—H9	0.9500
C4—N3	1.3367 (17)	C10—C11	1.384 (2)
C4—H4	0.9500	C10—H10	0.9500
C5—N3	1.4713 (17)	C11—H11	0.9500
C5—C6	1.5110 (18)	N1—N2	1.3092 (15)
C5—H5A	0.9900	N2—N3	1.3560 (15)
			
O1—C1—H1A	109.5	C11—C6—C5	120.62 (12)
O1—C1—H1B	109.5	C6—C7—C8	120.16 (15)
H1A—C1—H1B	109.5	C6—C7—H7	119.9
O1—C1—H1C	109.5	C8—C7—H7	119.9
H1A—C1—H1C	109.5	C9—C8—C7	120.57 (16)
H1B—C1—H1C	109.5	C9—C8—H8	119.7
O2—C2—O1	124.12 (13)	C7—C8—H8	119.7
O2—C2—C3	123.97 (12)	C8—C9—C10	119.48 (15)
O1—C2—C3	111.90 (10)	C8—C9—H9	120.3
N1—C3—C4	108.74 (11)	C10—C9—H9	120.3
N1—C3—C2	123.10 (11)	C9—C10—C11	120.74 (16)
C4—C3—C2	128.16 (11)	C9—C10—H10	119.6
N3—C4—C3	104.61 (11)	C11—C10—H10	119.6
N3—C4—H4	127.7	C6—C11—C10	120.32 (15)
C3—C4—H4	127.7	C6—C11—H11	119.8
N3—C5—C6	112.13 (11)	C10—C11—H11	119.8
N3—C5—H5A	109.2	N2—N1—C3	108.43 (10)
C6—C5—H5A	109.2	N1—N2—N3	107.32 (10)
N3—C5—H5B	109.2	C4—N3—N2	110.89 (11)
C6—C5—H5B	109.2	C4—N3—C5	129.62 (11)
H5A—C5—H5B	107.9	N2—N3—C5	119.49 (11)
C7—C6—C11	118.73 (13)	C2—O1—C1	115.14 (10)
C7—C6—C5	120.65 (12)		

## References

[bb1] Blessing, R. H. (1995). *Acta Cryst.* A**51**, 33–38. 10.1107/s0108767394005726 7702794

[bb2] Bruneau, C. & Dixneuf, P. H. (1999). *Acc. Chem. Res.* **32**, 311–323.

[bb3] Chen, C.-K., Tong, H.-C., Chen Hsu, C.-Y., Lee, C.-Y., Fong, Y. H., Chuang, Y.-S., Lo, Y.-H., Lin, Y.-C. & Wang, Y. (2009). *Organometallics*, **28**, 3358–3368.

[bb4] Cheng, C.-J., Tong, H.-C., Fong, Y.-H., Wang, P.-Y., Kuo, Y.-L., Lo, Y.-H. & Lin, C.-H. (2009). *Dalton Trans.* pp. 4435–4438. 10.1039/b901074m 19488439

[bb5] Farrugia, L. J. (1997). *J. Appl. Cryst.* **30**, 565.

[bb6] Farrugia, L. J. (1999). *J. Appl. Cryst.* **32**, 837–838.

[bb7] Krivopalov, V. P. & Shkurko, O. P. (2005). *Russ. Chem. Rev.* **74**, 339–379.

[bb8] Naota, T., Takaya, H. & Murahashi, S.-I. (1998). *Chem. Rev.* **98**, 2599–2660. 10.1021/cr9403695 11848973

[bb9] Nonius (1999). *COLLECT* Nonius BV, Delft, The Netherlands.

[bb10] Otwinowski, Z. & Minor, W. (1997). *Methods in Enzymology*, Vol. 276, *Macromolecular Crystallography*, Part A, edited by C. W. Carter Jr & R. M. Sweet, pp. 307–326. New York: Academic Press.

[bb11] Padwa, A. (1976). *Angew. Chem. Int. Ed. Engl.* **15**, 123–136.

[bb12] Sheldrick, G. M. (2008). *Acta Cryst.* A**64**, 112–122. 10.1107/S0108767307043930 18156677

[bb13] Trost, B. M., Toste, F. D. & Pinkerton, A. B. (2001). *Chem. Rev.* **101**, 2067–2096. 10.1021/cr000666b 11710241

